# Comparative performance of APACHE II, SOFA, and Charlson Comorbidity Index in sepsis mortality prediction

**DOI:** 10.17305/bb.2026.13931

**Published:** 2026-05-15

**Authors:** Srdjan Gavrilovic, Nikola Drincic Stipic, Milica Vasilic, Vladimir Andric, Djordje Nedeljkov, Vladimir Carapic

**Affiliations:** 1 Faculty of Medicine, University of Novi Sad, Novi Sad, Serbia; 2 Institute for Pulmonary Diseases of Vojvodina, Sremska Kamenica, Serbia

**Keywords:** Sepsis, septic shock, prognostic scores, APACHE II, SOFA, Charlson Comorbidity Index, mortality prediction, intensive care

## Abstract

Sepsis remains a leading cause of mortality in intensive care units (ICUs) globally; however, the effectiveness of conventional prognostic scoring systems varies across healthcare settings and patient populations. This study aimed to evaluate the comparative discriminative ability of the Acute Physiology and Chronic Health Evaluation II (APACHE II), Sequential Organ Failure Assessment (SOFA), and Charlson Comorbidity Index (CCI) in predicting 28-day mortality among critically ill septic patients. We conducted a retrospective observational study involving 392 consecutive adult patients diagnosed with sepsis according to the Sepsis-3 criteria, who were admitted to the ICU of a tertiary pulmonary care center in Serbia from January 2017 to December 2020. APACHE II scores were derived from the worst physiological values recorded within the first 24 hours of ICU admission; SOFA scores reflected the highest total score assessed during the same period; and CCI was calculated based on comorbidities present at admission. Discriminative performance was evaluated using receiver operating characteristic curve analysis. The overall 28-day mortality rate was 51.3%, which increased to 68.1% in patients with septic shock, with pneumonia being the source of infection in 97.4% of cases. APACHE II demonstrated the highest area under the curve (AUC) in the overall sepsis cohort (0.692, 95% CI 0.643–0.747), followed by SOFA (0.682, 95% CI 0.629–0.735) and CCI (0.667, 95% CI 0.613–0.720). In patients with septic shock, SOFA (AUC 0.671, 95% CI 0.561–0.782) and APACHE II (AUC 0.646, 95% CI 0.539–0.754) significantly outperformed CCI (AUC 0.423, 95% CI 0.302–0.543; *P* ═ 0.006 and *P* ═ 0.013, respectively), with no statistically significant difference between SOFA and APACHE II (*P* ═ 0.828). Optimal cut-off values were identified as SOFA ≥8, APACHE II ≥21, and CCI ≥4, with corresponding sensitivities of 59.7%, 67.7%, and 56.2%, respectively. Both APACHE II and SOFA exhibited modest and comparable discriminative abilities for mortality prediction in this high-severity population, while CCI demonstrated limited utility in cases of septic shock. These findings underscore the continued relevance of conventional scoring systems and highlight the necessity for population-specific validation in high-acuity settings.

## Introduction

Sepsis, defined as life-threatening organ dysfunction resulting from a dysregulated host response to infection, is a leading cause of morbidity and mortality in intensive care units (ICUs) worldwide [1–3]. Despite advancements in diagnostic and therapeutic strategies, the global burden of sepsis remains significant, with an estimated 166 million cases and 21.4 million sepsis-related deaths reported in 2021, accounting for 31.5% of total global deaths [3]. This burden is likely underestimated, particularly in low- and middle-income countries where diagnostic and reporting infrastructure is lacking [4, 5].

The heterogeneous nature of sepsis poses challenges in risk stratification and outcome prediction. Prognostic scoring systems are essential for assessing disease severity, guiding therapeutic decisions, benchmarking quality of care, and facilitating clinical research [6–8]. The Acute Physiology and Chronic Health Evaluation II (APACHE II), Sequential Organ Failure Assessment (SOFA), and Charlson Comorbidity Index (CCI) are the most extensively validated and widely used scoring systems in critical care [6, 7, 9]. Introduced in 1985, APACHE II incorporates acute physiological derangements, age, and chronic health status to predict hospital mortality [6]. Its accessibility, simplicity, and extensive validation have sustained its use despite the introduction of APACHE III and IV [10]. The SOFA score, designed specifically to assess organ dysfunction, gained prominence after its incorporation into the Sepsis-3 definition [1]. The CCI, originally intended to predict one-year mortality based on comorbidity burden, has proven useful for risk adjustment in critically ill patients [9]. However, the performance of prognostic models varies across healthcare settings, patient populations, and geographic regions [11–13]. Models developed in one country may require recalibration for application in another due to differences in healthcare systems, case mix, and clinical practices. Furthermore, most validation studies originate from high-income countries, with limited data from Central and Eastern Europe.

This study aims to evaluate and compare the discriminative performance of APACHE II, SOFA, and CCI in predicting 28-day mortality among critically ill patients with sepsis admitted to a tertiary-care medical ICU in Serbia, thereby providing region-specific validation data to inform clinical decision-making in similar healthcare contexts.

## Materials and methods

### Study design and setting

This retrospective observational cohort study was conducted at the ICU of the Clinic for Intensive Medicine and Pulmonary Vascular Diseases, Institute for Pulmonary Diseases of Vojvodina (IPBV), located in Sremska Kamenica, Serbia. This tertiary-care, university-affiliated medical ICU functions as a level 3 ICU according to Serbian national regulations and international classification systems, offering comprehensive invasive and noninvasive life support, serving as a regional referral center, and participating in critical care research and education [14, 15]. As a specialized pulmonary center, the ICU primarily admits patients with severe respiratory infections, resulting in a near-exclusive prevalence of pneumonia as the source of sepsis in this cohort.

### Study population

All consecutive adult patients (≥18 years) with sepsis admitted to the ICU between January 1, 2017, and December 31, 2020, were included. Of the 957 patients admitted during this period, 392 met all inclusion criteria and were enrolled in the final analysis. Sepsis was defined according to Sepsis-3 criteria as documented or suspected infection accompanied by an acute increase in SOFA score of ≥2 points from baseline [1, 16]. Baseline SOFA was assumed to be zero for patients admitted directly to the ICU from the emergency department or from outside the hospital. For patients transferred from another ward within the same institution or from another hospital, baseline SOFA was derived from available pre-transfer clinical documentation.

Septic shock was defined as sepsis requiring vasopressor therapy to maintain mean arterial pressure of ≥65 mmHg and lactate levels >2 mmol/L despite adequate fluid resuscitation [1, 17]. In this retrospective dataset, the adequacy of fluid resuscitation was determined based on the attending physician’s documented clinical judgment, supported by recorded fluid volumes administered prior to vasopressor initiation and explicit documentation of a fluid challenge in the medical record.

Pneumonia was diagnosed based on the attending physician’s clinical judgment, integrating clinical criteria (fever, leukocytosis, purulent respiratory secretions, worsening oxygenation), radiological findings (new or progressive infiltrate on chest X-ray or computed tomography [CT]), and microbiological data where available (sputum, bronchoalveolar lavage, or blood cultures). The cohort included both community-acquired and hospital-acquired pneumonia, reflecting the mixed admission pattern of our tertiary referral ICU.

Exclusion criteria included pregnancy or lactation, acute myocardial infarction, hemorrhagic or obstructive shock, cardiogenic pulmonary edema, requirement for urgent surgery within 6 h, ICU stay of <6 h (including early deaths), and confirmed SARS-CoV-2 infection (COVID-19).

### Data collection

Data were extracted from the integrated hospital information system (IBIS) and medical records. Infection was identified based on clinical documentation, including physician-recorded diagnoses, microbiological culture results where available (blood, respiratory, or urinary cultures), imaging findings, and initiation of antimicrobial therapy. In accordance with standard ICU protocols, vital signs were continuously monitored and recorded hourly for all patients. Laboratory measurements were ordered based on clinical need and institutional protocols. Collected variables included demographics (age, sex), admission diagnosis, sepsis source, presence of septic shock, vital signs (blood pressure, heart rate, respiratory rate, temperature, oxygen saturation, urine output), laboratory parameters (complete blood count, electrolytes, renal and hepatic function tests, arterial blood gases), Glasgow Coma Scale (GCS), comorbidities, mechanical ventilation requirement, and outcomes (ICU and hospital length of stay, 28-day and in-hospital mortality).

### Scoring systems

APACHE II was calculated using the worst physiological values within the first 24 h of ICU admission, along with chronic health and age points according to standard methodology [6]. Scores range from 0–71, with higher scores indicating greater severity. SOFA was calculated based on the worst values of six organ systems (respiratory, cardiovascular, hepatic, coagulation, renal, and neurological) assessed at each evaluation, with scores ranging from 0–24 [7]. In cases where the score was assessed multiple times within the first 24 h of ICU admission, the highest total score was used for analysis. For patients who were intubated and sedated at the time of assessment, the GCS score was recorded from clinical documentation obtained prior to intubation. If pre-intubation GCS was unavailable, a score of 15 (indicating the best possible response) was assigned, reflecting the assumption of intact neurological function before sedation, consistent with established practices in retrospective ICU studies. The CCI was calculated based on comorbidities present at admission according to standard methodology, with scores ranging from 0–33 [9].

### Outcomes

The primary outcome was 28-day all-cause mortality from ICU admission, while the secondary outcome was in-hospital mortality. Vital status at day 28 was determined through IBIS. For patients who remained hospitalized after ICU discharge, survival status was tracked through their continued in-hospital records. Patients discharged from the hospital prior to day 28 attended scheduled follow-up visits at the same institution, where vital status was confirmed.

### Ethical statement

The study was approved by the Ethics Committee of the IPBV (approval number: 12-V/2, date: May 19, 2022) and conducted in accordance with the Declaration of Helsinki. Given its retrospective nature and the use of de-identified data, the requirement for individual informed consent was waived.

### Statistical analysis

Statistical analyses were performed using International Business Machines Statistical Package for the Social Sciences (IBM SPSS) Statistics version 23.0 (IBM Corp., Armonk, NY) and Python (version 3.14). There were no missing values in any key variables used for score calculation; thus, the analysis was conducted on the complete dataset (complete-case analysis).

Continuous variables were assessed for normality using the Shapiro-Wilk test and presented as mean ± standard deviation (SD) or median with interquartile range (IQR), as appropriate. Categorical variables were expressed as frequencies and percentages.

Between-group comparisons were conducted using Student’s *t*-test or Mann-Whitney *U* test for continuous variables and one-sample chi-square or chi-square of independence for categorical variables. The discriminative performance of each scoring system was evaluated using receiver operating characteristic (ROC) curve analysis, calculating the area under the curve (AUC) and 95% confidence intervals (CIs). Optimal cut-off values were determined using Youden’s index, with calculations of sensitivity, specificity, positive predictive value (PPV), negative predictive value (NPV), likelihood ratios, and overall accuracy. Comparisons of AUCs were performed using DeLong’s test, with CIs for AUCs estimated using 1000 bootstrap resamples. Overall predictive performance of the models was assessed using the Brier score, calculated as the mean squared difference between predicted probabilities and observed binary outcomes. Model calibration was evaluated using the Hosmer-Lemeshow goodness-of-fit test, with *P* > 0.05 indicating acceptable calibration. Given the retrospective nature of the study, a formal a priori sample size calculation was not performed; however, a post-hoc power analysis for ROC curve analysis was conducted using the Hanley-McNeil method to confirm adequacy of sample size. Statistical significance was set at *P* < 0.05 (two-tailed).

## Results

### Patient characteristics

A total of 392 patients meeting the inclusion criteria were enrolled in the study. The cohort was predominantly male (57.7%), with a median age of 63 years (IQR 52–71). Pneumonia accounted for 97.4% of the identified infection sources. Invasive mechanical ventilation was required for 90.1% of patients within the first 24 h of ICU admission, with an additional 3.1% receiving noninvasive respiratory support. Baseline demographic, clinical, and comorbidity characteristics are summarized in Table 1.

**Table 1:** Baseline characteristics of patients with sepsis according to 28-day mortality

**Variable**	**Overall** **(*n* ═ 392)**	**Survivors (*n* ═ 191)**	**Non-survivors (*n* ═ 201)**	***P* value**
**Demographics**				
Age, years - median (IQR)	63 (52--71)	60 (48--68)	65 (56--73)	**<0.001**
Male sex - *n* (%)	226 (57.7%)	107 (56.0%)	119 (59.2%)	ns
Female sex - *n* (%)	166 (42.3%)	84 (44.0%)	82 (40.8%)	ns
**Sepsis characteristics**				
Septic shock - *n* (%)	116 (29.6%)	37 (19.4%)	79 (39.3%)	**<0.001**
Pneumonia - *n* (%)	382 (97.4%)	185 (96.9%)	197 (98.0%)	ns
Urosepsis - *n* (%)	10 (2.6%)	6 (3.1%)	4 (2.0%)	ns
Invasive mechanical ventilation - *n* (%)	353 (90.1%)	-	-	-
Noninvasive respiratory support (NIV/HFNC) - *n* (%)	12 (3.1%)	-	-	-
**Laboratory parameters**				
Lactate, mmol/L - median (IQR)	2.37 (1.60--3.92)	2.00 (1.49--3.32)	2.84 (1.72--4.68)	**<0.001**
**Comorbidities (Charlson Index components)**				
Age ≥65 years - *n* (%)	179 (45.7%)	71 (37.2%)	108 (53.7%)	**<0.001**
Congestive heart failure - *n* (%)	84 (21.4%)	27 (14.1%)	57 (28.4%)	**0.001**
Chronic lung disease - *n* (%)	87 (22.2%)	30 (15.7%)	57 (28.4%)	**0.004**
Leukemia / myeloma - *n* (%)	21 (5.4%)	5 (2.6%)	16 (8.0%)	**0.016**
Myocardial infarction - *n* (%)	32 (8.2%)	16 (8.4%)	16 (8.0%)	ns
Cerebrovascular disease - *n* (%)	30 (7.7%)	16 (8.4%)	14 (7.0%)	ns
Diabetes mellitus - *n* (%)	12 (3.1%)	5 (2.6%)	7 (3.5%)	ns
Chronic kidney disease - *n* (%)	14 (3.6%)	6 (3.1%)	8 (4.0%)	ns
Chronic liver disease - *n* (%)	18 (4.6%)	8 (4.2%)	10 (5.0%)	ns
Peripheral vascular disease - *n* (%)	15 (3.8%)	6 (3.1%)	9 (4.5%)	ns
Connective tissue disease - *n* (%)	14 (3.6%)	7 (3.7%)	7 (3.5%)	ns
Dementia - *n* (%)	13 (3.3%)	8 (4.2%)	5 (2.5%)	ns
Hemiplegia - *n* (%)	11 (2.8%)	5 (2.6%)	6 (3.0%)	ns
Lymphoma - *n* (%)	7 (1.8%)	3 (1.6%)	4 (2.0%)	ns
Malignant tumor within 5 years - *n* (%)	5 (1.3%)	1 (0.5%)	4 (2.0%)	ns
AIDS - *n* (%)	4 (1.0%)	1 (0.5%)	3 (1.5%)	ns
**Outcomes**				
28-day mortality - *n* (%)	201 (51.3%)	-	-	-
In-hospital mortality - *n* (%)	211 (53.8%)	-	-	-
ICU length of stay, days - median (IQR)	5 (2--10)	6 (3--12)	4 (2--9)	**<0.01**
Hospital length of stay, days - median (IQR)	15 (7--26)	23 (15--32)	8 (4--15)	**<0.001**

*Continuous variables are presented as median (IQR) following Shapiro-Wilk normality testing. Between-group comparisons were conducted using the Mann-Whitney *U* test for continuous variables and the chi-square test of independence for categorical variables. *P* values highlighted in bold indicate statistical significance (*P* < 0.05). Abbreviations: AIDS, acquired immunodeficiency syndrome; HFNC, high-flow nasal cannula; ICU, intensive care unit; IQR, interquartile range; n, number; NIV, noninvasive ventilation; ns, not significant.

### Mortality outcomes

The 28-day mortality rate was 51.3% (201/392) in the overall cohort, with a rate of 68.1% (79/116) among patients with septic shock, compared to 44.2% (122/276) in those without shock (*P* < 0.001). In-hospital mortality was 53.8% (211/392) overall and 71.6% (83/116) in patients with septic shock. Septic shock was significantly associated with both 28-day (χ^2^ ═ 17.730, *P* < 0.001) and in-hospital mortality (χ^2^ ═ 19.826, *P* < 0.001).

### Prognostic score performance

**Figure 1.**
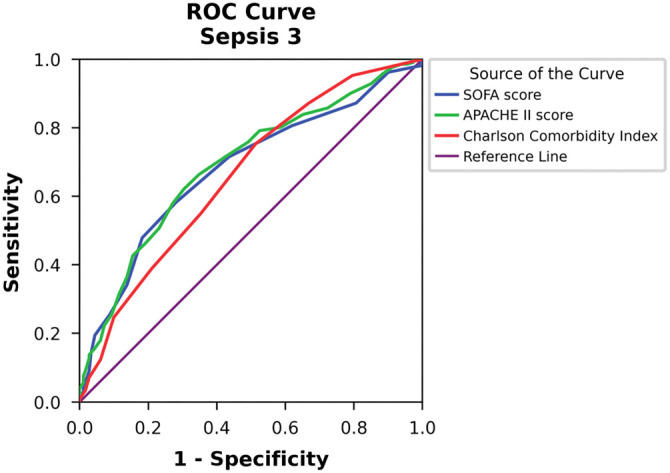
**Discriminatory performance of SOFA, APACHE II, and CCI for predicting 28-day mortality in patients with sepsis.** Receiver operating characteristic curves comparing the prognostic accuracy of SOFA, APACHE II, and CCI for 28-day mortality prediction in the overall sepsis cohort. Scores were calculated using data obtained within the first 24 h of ICU admission. APACHE II showed the highest discriminatory performance (AUC 0.692, 95% CI 0.643–0.747), followed by SOFA (AUC 0.682, 95% CI 0.629–0.735) and CCI (AUC 0.667, 95% CI 0.613–0.720). The diagonal reference line represents no discriminatory ability. Abbreviations: APACHE II, Acute Physiology and Chronic Health Evaluation II; AUC, area under the curve; CCI, Charlson Comorbidity Index; CI, confidence interval; ICU, intensive care unit; ROC, receiver operating characteristic; SOFA, Sequential Organ Failure Assessment.

**Figure 2.**
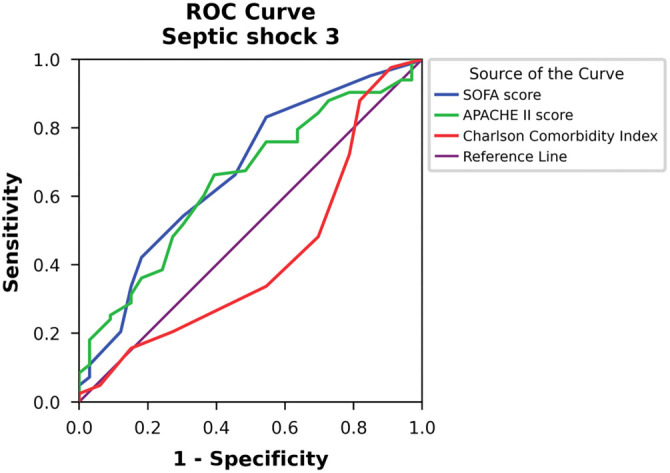
**Discriminatory performance of SOFA, APACHE II, and CCI for predicting 28-day mortality in patients with septic shock.** Receiver operating characteristic curves comparing the prognostic accuracy of SOFA, APACHE II, and CCI for 28-day mortality prediction in the septic shock subgroup. Scores were calculated using data obtained within the first 24 h of ICU admission. SOFA showed the highest discriminatory performance (AUC 0.671, 95% CI 0.561–0.782), followed by APACHE II (AUC 0.646, 95% CI 0.539–0.754), whereas CCI demonstrated poor discriminatory ability (AUC 0.423, 95% CI 0.302–0.543). The diagonal reference line indicates no discriminatory ability. Abbreviations: APACHE II, Acute Physiology and Chronic Health Evaluation II; AUC, area under the curve; CCI, Charlson Comorbidity Index; CI, confidence interval; ICU, intensive care unit; ROC, receiver operating characteristic; SOFA, Sequential Organ Failure Assessment.

The performance of the SOFA, APACHE II scores, and CCI in patients with sepsis and septic shock, in relation to 28-day mortality, is presented in Table 2. Table 3 displays the apparent and optimism-corrected (bootstrap) AUC values, Brier scores, and pairwise DeLong comparisons for each score, while Figures 1 and 2 illustrate the corresponding ROC curves for patients with sepsis and septic shock, respectively.

**Table 2:** SOFA and APACHE II scores, and CCI in sepsis and septic shock patients based on 28-day mortality rates

**Group**	**Mean#**	**SD**	**Min**	**Max**	**Median**	**P25--P75**	**Significance‡**
**SOFA**							
Sepsis^a^ (*n* ═ 392)							
Non-survivors	8.59***	3.39	2	19	8	6--11	
Survivors	6.61	2.64	2	16	6	5--8	
Total	7.62	3.2	2	19	7	5--9	
Septic Shock^b^ (*n* ═ 116)							
Non-survivors	11.20**	2.86	5	19	11	9--13	b/a***
Survivors	9.27	2.76	5	16	9	7--11	b/a***
Total	10.59	2.96	5	19	10	8.25--13	b/a***
**APACHE II**							
Sepsis^a^ (*n* ═ 392)							
Non-survivors	24.06***	7.66	7	47	24	19--29	
Survivors	18.87	6.8	3	38	18	14--23	
Total	21.53	7.7	3	47	21	16--27	
Septic Shock^b^ (*n* ═ 116)							
Non-survivors	27.66**	7.31	12	47	27	23--34	b/a**
Survivors	23.05	6.86	8	38	23	17--28.5	b/a**
Total	26.19	7.46	8	47	26	20.25--31.75	b/a***
**CCI**							
Sepsis^a^ (*n* ═ 392)							
Non-survivors	4.06***	2.2	0	10	4	3--6	
Survivors	2.74	2.17	0	9	3	1--4	
Total	3.42	2.79	0	10	3	2--5	
Septic Shock^b^ (*n* ═ 116)							
Non-survivors	4.03 ns	2.26	0	10	5	2--5	
Survivors	4.05	2.46	0	9	4	2.5--5.5	
Total	4.03	2.32	0	10	4	2--5	b/a*

#Mann-Whitney *U* test by mortality; ^‡^ Mann-Whitney *U* test vs. sepsis; a, overall sepsis cohort; b, septic shock subgroup; b/a, statistical comparison between the septic shock subgroup (b) and the overall sepsis cohort (a); ^*^ *P* < 0.05; ^**^ *P* < 0.01; ^***^ *P* < 0.001. Abbreviations: APACHE II, Acute Physiology and Chronic Health Evaluation II; CCI, Charlson Comorbidity Index; Max, maximum; Min, minimum; n, number; ns, not significant; P25–P75, 25th–75th percentile; SD, standard deviation; SOFA, Sequential Organ Failure Assessment.

For the APACHE II score, mean values were significantly higher in non-survivors compared to survivors (24.06 ± 7.66 vs. 18.87 ± 6.80, *P* < 0.001). In septic shock patients, the scores were 27.66 ± 7.31 for non-survivors vs 23.05 ± 6.86 for survivors (*P* < 0.01). ROC analysis demonstrated an AUC of 0.692 (95% CI 0.643–0.747, *P* < 0.001) for patients with sepsis (Tables 2 and 3, Figure 1) and 0.646 (95% CI 0.539–0.754, *P* ═ 0.014) for those with septic shock (Tables 2 and 3, Figure 2).

For the SOFA score, mean values were 8.59 ± 3.39 in non-survivors vs 6.61 ± 2.64 in survivors (*P* < 0.001). In the septic shock group, scores were 11.20 ± 2.86 vs 9.27 ± 2.76 (*P* < 0.01). The AUC was 0.682 (95% CI 0.629–0.735, *P* < 0.001) for patients with sepsis (Tables 2 and 3, Figure 1) and 0.671 (95% CI 0.561–0.782, *P* ═ 0.004) for those with septic shock (Tables 2 and 3, Figure 2).

Regarding the CCI, mean CCI values were 4.06 ± 2.20 in non-survivors vs 2.74 ± 2.17 in survivors (*P* < 0.001). In septic shock patients, the difference was not significant (4.03 ± 2.26 vs. 4.05 ± 2.46, *P* > 0.05). The AUC was 0.667 (95% CI 0.613–0.720, *P* < 0.001) for patients with sepsis (Tables 2 and 3, Figure 1) but only 0.423 (95% CI 0.302–0.543, *P* ═ 0.195) for those with septic shock (Tables 2 and 3, Figure 2).

The corresponding Brier scores were 0.225 for SOFA, 0.221 for APACHE II, and 0.230 for CCI for patients with sepsis, and 0.196, 0.202, and 0.243, respectively, for patients with septic shock (Table 3). Hosmer-Lemeshow testing demonstrated acceptable calibration for all three scores in both the overall sepsis cohort and the septic shock subgroup (all *P* > 0.05; range 0.206–0.893). Post-hoc power analysis confirmed adequate statistical power for all scores in the sepsis cohort (>99%) and for SOFA and APACHE II in the septic shock subgroup (95.5% and 87.0%, respectively).

### Optimal cut-off values

Table 4 displays the 2×2 contingency data, while Table 5 summarizes the corresponding diagnostic performance.

For the overall sepsis cohort, optimal cut-off values were: SOFA ≥ 8 (sensitivity 59.7%, specificity 71.7%, accuracy 65.6%, odds ratio [OR] 3.76, 95% CI 2.46–5.74), APACHE II ≥ 21 (sensitivity 67.7%, specificity 64.9%, accuracy 66.3%, OR 3.87, 95% CI 2.55–5.89), and CCI ≥ 4 (sensitivity 56.2%, specificity 64.9%, accuracy 60.5%, OR 2.38, 95% CI 1.58–3.57). In the septic shock cohort, SOFA ≥ 8 achieved a sensitivity of 91.1% and specificity of 32.4% (accuracy 72.4%, OR 4.94, 95% CI 1.75–13.93), while APACHE II ≥ 25 demonstrated a sensitivity of 68.4% and specificity of 62.2% (accuracy 66.4%, OR 3.55, 95% CI 1.57–8.03). CCI ≥ 4 showed a sensitivity of 50.6% and specificity of 37.8% (accuracy 46.6%, OR 0.62, 95% CI 0.28–1.39) (Tables 4 and 5).

**Table 3:** Discriminatory performance of three prognostic scores in sepsis and septic shock

	**Apparent AUC (95% CI)**	***P* vs 0.5**	**Optimism-corrected AUC (95% CI)**	***P* (DeLong); AUC difference (95% CI)**	**Brier score**
**Sepsis (*n* ═ 392)**					
SOFA	0.682 (0.629--0.735)	*P* < 0.001	0.682 (0.631--0.734)	*P* ═ 0.608; –0.013 (–0.062 to 0.035) [SOFA vs APACHE II]	0.225
APACHE II	0.692 (0.643--0.747)	*P* < 0.001	0.695 (0.644--0.747)	*P* ═ 0.382; 0.029 (–0.035 to 0.093) [APACHE II vs CCI]	0.221
CCI	0.667 (0.613--0.720)	*P* < 0.001	0.667 (0.615--0.717)	*P* ═ 0.671; 0.015 (–0.059 to 0.086) [SOFA vs CCI]	0.230
**Septic shock (*n* ═ 116)**					
SOFA	0.671 (0.561--0.782)	0.004	0.679 (0.578--0.792)	*P* ═ 0.828; 0.010 (–0.085 to 0.097) [SOFA vs APACHE II]	0.196
APACHE II	0.646 (0.539--0.754)	0.014	0.649 (0.566--0.789)	***P* ═ 0.013;** **0.205 (0.056 to 0.352) [APACHE II vs CCI]**	0.202
CCI	0.423 (0.302--0.543)	0.195	0.424 (0.325--0.557)	***P* ═ 0.006;** **0.215 (0.043 to 0.380)** **[SOFA vs CCI]**	0.243

*Optimism-corrected AUC values were derived through bootstrap resampling (1,000 iterations) to mitigate the risk of overfitting. The notation *P* vs 0.5 indicates the significance of each AUC in relation to the null hypothesis of random discrimination. The *P* (DeLong) values represent pairwise comparisons of individual scores utilizing DeLong’s test for correlated ROC curves. The AUC difference is calculated as Score 1 minus Score 2, as specified. Additionally, 95% CIs were estimated using bootstrap resampling (1,000 iterations). Bold *P* values denote statistically significant differences (*P* < 0.05). Abbreviations: APACHE II, Acute Physiology and Chronic Health Evaluation II; AUC, area under the curve; CCI, Charlson Comorbidity Index; CI, confidence interval; n, number; ROC, receiver operating characteristic; SOFA, Sequential Organ Failure Assessment.

**Table 4:** Distribution of sepsis and septic shock patients based on SOFA score, APACHE II score, and CCI cut-offs, alongside 28-day mortality and corresponding ORs

**Condition**	**Cut-off**	**Deceased**†	**Survivors†**	**Total**	***P*-value**	**OR (95% CI)**
**Sepsis**						
SOFA	≥8	120	54	174	**<0.001**	**3.76 (2.46--5.74)**
	≤7	81	137	218		
APACHE II	≥21	136	67	203	**<0.001**	**3.87 (2.55--5.89)**
	≤20	65	124	189		
CCI	≥4	113	67	180	**<0.001**	**2.38 (1.58--3.57)**
	≤3	88	124	212		
**Septic shock**						
SOFA	≥8	72	25	97	**0.003**	**4.94 (1.75--13.93)**
	≤7	7	12	19		
APACHE II	≥25	54	14	68	**0.004**	**3.55 (1.57--8.03)**
	≤24	25	23	48		
CCI	≥4	40	23	63	0.336	0.62 (0.28--1.39)
	≤3	39	14	53		

*ORs were calculated using the Woolf method (log-odds). Bold values indicate statistical significance (*P* < 0.05), with cut-off values determined by the maximum Youden’s index. † Number of patients; chi-square test of independence (Pearson, without Yates’ correction); all expected cell frequencies were ≥ 5. Abbreviations: APACHE II, Acute Physiology and Chronic Health Evaluation II; CCI, Charlson Comorbidity Index; CI, confidence interval; OR, odds ratio; SOFA, Sequential Organ Failure Assessment.

**Table 5:** Diagnostic performance at the optimal cut-off values in sepsis and septic shock

		**Sepsis**			**Septic shock**
	**SOFA**	**APACHE II**	**CCI**	**SOFA**	**APACHE II**	**CCI**
Cut-off	7.5	20.5	3.5	7.5	24.5	3.5
J	0.314	0.326	0.211	0.235	0.306	–0.116
Se	0.597	0.677	0.562	0.911	0.684	0.506
Sp	0.717	0.649	0.649	0.324	0.622	0.378
PPV	0.690	0.670	0.628	0.742	0.794	0.635
NPV	0.628	0.656	0.585	0.632	0.479	0.264
LR+	2.112	1.929	1.603	1.349	1.807	0.814
LR+ (95% CI)	1.638–2.716	1.555–2.392	1.274–2.012	1.067–1.702	1.166–2.809	0.583–1.134
LR--	0.562	0.498	0.674	0.273	0.509	1.305
LR-- (95% CI)	0.465–0.680	0.397–0.624	0.559–0.815	0.118–0.640	0.337–0.766	0.817–2.090
Acc	0.656	0.663	0.605	0.724	0.664	0.466

*95% CIs for likelihood ratios were calculated using the log transformation method. Cut-off values were determined based on the maximum Youden’s index. Abbreviations: Acc, accuracy; APACHE II, Acute Physiology and Chronic Health Evaluation II; CCI, Charlson Comorbidity Index; CI, confidence interval; J, Youden’s index; LR+, positive likelihood ratio; LR--, negative likelihood ratio; NPV, negative predictive value; PPV, positive predictive value; Se, sensitivity; SOFA, Sequential Organ Failure Assessment; Sp, specificity.

### Impact of comorbidities

Among all sepsis patients, significantly higher 28-day mortality was observed in those aged ≥65 years with congestive heart failure, chronic lung disease, or leukemia/myeloma. In septic shock patients, only age ≥65 years and congestive heart failure remained significant (Table 1).

## Discussion

### Principal findings

This study evaluated the discriminative performance of three widely used prognostic scoring systems in critically ill patients with sepsis at a tertiary care medical ICU in Serbia. Our principal findings indicate that the APACHE II score exhibited a marginally higher AUC in the overall sepsis cohort (0.692 vs. 0.682), though without a statistically significant difference from the SOFA score (*P* ═ 0.608; AUC difference 0.013, 95% CI --0.062 to 0.035). In septic shock patients, SOFA (AUC 0.671, 95% CI 0.561–0.782) and APACHE II (AUC 0.646, 95% CI 0.539–0.754) demonstrated comparable discriminative abilities with no statistically significant difference between them (*P* ═ 0.828), while both significantly outperformed the CCI (AUC 0.423; *P* ═ 0.006 and *P* ═ 0.013, respectively). The wide CIs in the septic shock subgroup reflect the smaller sample size and warrant cautious interpretation. The CCI exhibited acceptable discrimination in general sepsis (AUC 0.667) but performed at chance level in septic shock.

### Contextualizing mortality rates

The 28-day mortality rate of 51.3% in our overall cohort, rising to 68.1% in septic shock, is substantially higher than reported in recent multicenter studies from high-income countries (25%–35% for sepsis, 35%–50% for septic shock) [18, 19]. Several factors likely contribute to this discrepancy. First, our study was conducted in a specialized medical ICU serving as a tertiary referral center with an adjacent intermediate care unit. This organizational structure leads to the selective admission of the most critically ill patients to the ICU, as evidenced by the exceptionally high proportion (90.1%) requiring invasive mechanical ventilation within the first 24 h and the high prevalence of multiple organ dysfunction. This selection bias toward severity parallels findings by Peelen et al., who reported higher ICU mortality in Dutch hospitals with step-down units compared to those without, attributable to case mix differences [20]. Second, pneumonia, the predominant infection source in 97.4% of our patients, is associated with higher mortality than other sources of infection [17, 21]. Our patient population was also elderly (median age 63 years) with a substantial comorbidity burden. Third, regional healthcare system factors may play a role. Similar mortality rates have been reported from other Central and Eastern European centers using comparable definitions and settings (Croatian medical ICU: 72.1% septic shock mortality; North Macedonian ICU: 71.4%) [22, 23]. While our single-center design limits generalizability to national sepsis mortality, these findings align with broader patterns of elevated critical illness mortality in the region, potentially reflecting differences in pre-hospital care, referral patterns, resource availability, and population health status.

### Comparative performance of scoring systems

The discriminative ability of APACHE II and SOFA in our study (AUCs 0.692 and 0.682, respectively) is lower than reported in large multicenter databases (typically 0.75–0.85) [24–26]. However, this finding is methodologically anticipated and consistent with fundamental principles of prognostic model performance. Prognostic scores demonstrate optimal discrimination in heterogeneous populations with a wide range of illness severity and mortality rates, typically between 10%–40% [27]. In our highly selected population with a 51% mortality rate and near-universal mechanical ventilation, most patients clustered in the high-severity range, which inherently limits discriminative capacity. This “ceiling effect” has been well-documented: as population homogeneity and mortality increase, AUC values necessarily decline, even for well-calibrated models [27, 28].

The recently published Sequential Organ Failure Assessment 2 (SOFA-2) development study by Ranzani et al., which analyzed over 3.3 million ICU admissions across nine countries, provides essential validation for our findings. In their diverse cohort, which exhibited an ICU mortality rate of 8.1% (ranging from 4.5% to 20.5%), the original SOFA score (SOFA-1) demonstrated an AUC of 0.77 (95% CI 0.74–0.81). In contrast, the updated SOFA-2 score achieved an AUC of 0.79 (95% CI 0.76–0.81), reflecting a modest improvement of 0.02 [28]. Our SOFA AUC of 0.682, derived from a cohort with a mortality rate of 51%, represents a proportionally expected decline in discriminative performance due to the significantly higher baseline risk and disease severity in our population. To further contextualize this, Ranzani’s study included cohorts with mortality rates ranging from 4.5% to 20.5%, while our cohort’s mortality exceeded 50% [28]. The established relationship between baseline mortality and discriminative capacity is underscored by their finding that SOFA-2 performance varied significantly across cohorts based on case mix characteristics. Therefore, our lower AUC reflects appropriate score behavior in a high-severity population rather than poor performance. The Brier scores corroborate this interpretation, with values ranging from 0.221–0.230 for the overall sepsis cohort and 0.196–0.243 for septic shock, indicating modest yet acceptable predictive accuracy consistent with the high-severity, low-variability characteristics of this population.

Regarding the comparative performance of individual scores, although SOFA exhibited a numerically higher AUC than APACHE II in septic shock (0.671 vs. 0.646), this difference was not statistically significant (DeLong *P* ═ 0.828; AUC difference 0.010, 95% CI --0.085 to 0.097), and the overlapping CIs (SOFA: 0.561–0.782; APACHE II: 0.539–0.754) preclude any claims of superiority. Both scores demonstrated comparable discriminative ability in this subgroup. In contrast, both SOFA and APACHE II showed significantly better discrimination than CCI in septic shock, with AUC differences of 0.215 (95% CI 0.043–0.380) and 0.205 (95% CI 0.056–0.352), respectively. Nevertheless, the theoretical framework underlying SOFA, which includes dynamic organ dysfunction assessment and explicit incorporation of vasopressor requirements, may render it particularly relevant in the context of septic shock, where catecholamine-resistant hypotension characterizes the syndrome. Similarly, although APACHE II demonstrated a numerically higher AUC in the overall sepsis cohort (0.692 vs. 0.682), this difference was also not statistically significant (*P* ═ 0.608), consistent with its comprehensive physiological assessment that includes acute derangements, age, and chronic health status.

While no formal combined model analysis was conducted, the differing discriminative performances of APACHE II, SOFA, and CCI across sepsis severity levels suggest a potentially complementary prognostic value rather than redundancy. Future prospective studies are necessary to formally evaluate the combined score performance to substantiate this interpretation.

### Comorbidity assessment in sepsis prognostication

Although APACHE II, SOFA, and CCI evaluate fundamentally different constructs, this heterogeneity is a methodological strength that allows for the assessment of the relative contributions of acute physiological derangement and chronic comorbidity burden to mortality prediction across the spectrum of sepsis severity. The CCI exhibited acceptable performance in overall sepsis (AUC 0.667) but demonstrated poor discrimination in septic shock (AUC 0.423). The AUC of 0.423 for CCI in septic shock necessitates specific explanation. Statistically, this value does not significantly differ from random chance (*P* ═ 0.195, 95% CI 0.302–0.543), as the CI crosses the 0.5 threshold. This finding reflects nearly identical CCI values between non-survivors and survivors in the septic shock subgroup (4.03 ± 2.26 vs. 4.05 ± 2.46, *P* > 0.05). When score values are nearly indistinguishable between outcome groups, the AUC approximates 0.5 by definition, and random sampling variation in a relatively small subgroup (*N* ═ 116) can yield values slightly below this threshold. This finding may suggest that in the most severely ill patients, acute physiological derangement rather than chronic comorbidity burden becomes the primary determinant of short-term mortality risk. However, this interpretation should be approached with caution due to the limited subgroup size and wide CIs observed. Our findings are consistent with those of Ladha et al., who reported similar CCI performance for 30-day mortality (AUC 0.65) among heterogeneous ICU patients, with notably improved prediction for longer-term outcomes (one-year AUC 0.72) [29]. This pattern indicates that comorbidity indices are most valuable for predicting outcomes where baseline health status plays a more significant role relative to acute illness severity.

In our study, the significant association between specific comorbidities (age ≥65 years, heart failure, chronic lung disease, hematological malignancy) and mortality underscores that baseline health status remains relevant even in critically ill populations, consistent with extensive literature [30–32]. However, the limited utility of CCI in septic shock suggests that comorbidity indices should complement rather than replace acute physiology-based scores in the most critically ill patients. This observation has practical implications for score selection and interpretation. In patients presenting with sepsis without shock, where mortality risk is more heterogeneous and physiological derangements are less severe, incorporating comorbidity assessment (via CCI or the chronic health component of APACHE II) provides meaningful prognostic information. This likely accounts for APACHE II’s marginally higher AUC than SOFA in our overall sepsis cohort (0.692 vs. 0.682), albeit without statistical significance (*P* ═ 0.608). Conversely, in septic shock, where acute organ dysfunction predominates, acute physiology scores (SOFA, APACHE II physiological component) capture the primary determinants of outcomes, and adding CCI contributes minimal incremental value for short-term mortality prediction.

### Clinical implications

Despite lower AUCs than those observed in large heterogeneous populations, our findings support the continued clinical utility of APACHE II and SOFA in high-acuity settings, with CCI providing valuable complementary information in appropriate contexts. The identified cut-off values were derived and validated within the same cohort and should be regarded as exploratory rather than definitive thresholds. The established cut-off values identified patients with significantly elevated mortality risk: SOFA ≥ 8 (OR 3.76, 95% CI 2.46–5.74), APACHE II ≥ 21 (OR 3.87, 95% CI 2.55–5.89), and CCI ≥ 4 (OR 2.38, 95% CI 1.58–3.57) in the overall sepsis cohort, and SOFA ≥ 8 (OR 4.94, 95% CI 1.75–13.93) and APACHE II ≥ 25 (OR 3.55, 95% CI 1.57–8.03) in septic shock. CCI ≥ 4 did not significantly discriminate mortality in septic shock (OR 0.62, 95% CI 0.28–1.39), consistent with its poor discriminative performance in this subgroup (Table 4). These thresholds can inform bedside prognostication, family counseling, and clinical trial stratification, demonstrating a reasonable balance between sensitivity and specificity (approximately 60%–70% for acute scores), although clinicians should interpret them alongside other clinical factors and the trajectory of illness. It is important to note that PPV and NPV are strongly influenced by the prevalence of the outcome in the studied population. In our cohort, with a 28-day mortality rate of 51.3%, substantially higher than in most published sepsis series, PPV values are likely inflated, while NPV values may be deflated compared to what would be observed in lower-mortality settings. Clinicians applying these cut-offs in populations with different baseline mortality should therefore emphasize the prevalence-independent likelihood ratios (LR+ and LR--) when estimating post-test probability of death, and exercise caution when generalizing PPV and NPV values beyond this specific clinical context. LR+ values ranged from 1.60–2.11 in the overall sepsis cohort, indicating modest yet meaningful positive shifts in post-test probability, while LR-- values ranged from 0.50–0.67, suggesting limited ability to rule out mortality when scores fall below the cut-off.

The differential performance of CCI across sepsis severity levels has significant practical implications. In patients presenting with sepsis without shock, incorporating comorbidity assessment adds valuable prognostic information and may guide discussions about treatment intensity, goals of care, and realistic outcome expectations based on both acute illness severity and baseline health status. However, in septic shock, where acute organ dysfunction predominates, clinicians should prioritize acute physiology scores (APACHE II, SOFA) for immediate risk stratification and clinical decision-making, while recognizing that chronic comorbidity burden may become more relevant for longer-term prognostication and post-ICU trajectory.

The finding that conventional scores maintain acceptable performance even in severely ill populations is both reassuring and practically relevant. While newer machine learning models and disease-specific scores may provide incremental improvements, the simplicity, familiarity, and cost-free implementation of APACHE II, SOFA, and CCI advocate for their continued use, particularly in resource-limited settings. The complementary nature of these scores—capturing acute physiological disturbance, organ dysfunction trajectory, and chronic disease burden—suggests that an integrated assessment using multiple tools may yield more comprehensive prognostic information across the spectrum of sepsis severity.

### Need for local validation and regional registries

Our results underscore the critical need for local validation of prognostic models prior to clinical implementation, a principle emphasized by numerous authorities in critical care outcomes research [33–35]. Variations in healthcare systems, patient demographics, and clinical practices necessitate region-specific performance assessments and, when appropriate, recalibration. Local validation of discriminatory performance is essential for reliable risk adjustment and meaningful interpretation of inter-ICU comparisons. This is particularly important in high-mortality ICUs and homogeneous disease cohorts, where limited variability in disease severity may constrain AUC values due to well-documented “ceiling effects” [27, 28, 36, 37]. We propose that center- and disease-specific AUC reporting should be viewed as a marker of methodological rigor and high standards in intensive care practice, rather than a limitation of prognostic modeling. Neglecting local performance metrics may obscure significant differences in case mix or quality of care that could impact the validity of benchmarking exercises.

The high mortality rates observed in our study and similar centers in Central and Eastern Europe highlight the necessity for national ICU registries to provide robust epidemiological data, enable benchmarking, support quality improvement initiatives, and facilitate the development of prognostic models tailored to regional populations [38, 39]. Furthermore, successful implementation of resource-intensive registries in countries such as Sri Lanka, Brazil, and Pakistan demonstrates feasibility in middle-income settings [38–40].

Research evaluating prognostic scores in Serbian sepsis patients is limited. Djikic et al. assessed six scoring systems for predicting 24-hour mortality in septic patients presenting to the emergency department, focusing primarily on early emergency care rather than ICU outcomes [41]. To our knowledge, this study represents the first evaluation of multiple prognostic scores specifically in ICU-admitted sepsis patients according to the Sepsis-3 definition in Serbia, highlighting a significant gap in regional critical care outcomes research. A national registry would not only provide the infrastructure for rigorous local validation but also enable participation in international collaborative research, such as the SOFA-2 development consortium, ensuring that regional populations are adequately represented in the next generation of prognostic tools.

### Future directions

The recent publication of SOFA-2 in 2025 marks a significant milestone in critical care outcome measurement and offers a clear roadmap for future research. Priority areas include multicenter studies encompassing diverse ICU types and healthcare settings within the region, external validation of contemporary scoring systems in Central and Eastern European populations, investigation of dynamic scores that incorporate illness trajectory during the ICU stay, assessment of machine learning models trained on regional data, and evaluation of how scoring systems correlate with long-term functional outcomes and quality of life beyond acute mortality.

Establishing a national ICU registry remains crucial, providing the infrastructure for robust outcomes research, quality benchmarking, participation in international validation studies, and the development of regionally optimized prognostic models that accurately reflect local case mix and resource availability.

### Limitations

This study has several important limitations. The single-center design in a tertiary medical ICU limits generalizability to other settings, particularly surgical ICUs, community hospitals, and healthcare systems with different organizational structures. The highly selective, severely ill population, characterized by near-universal mechanical ventilation and multiple organ dysfunction, inherently limits score discrimination and may not reflect broader sepsis populations.

The retrospective observational design precludes causal inference and does not permit comprehensive adjustment for all confounders. Scoring was based on the worst 24-hour values, which is standard for research but not intended for real-time decision-making and may miss early clinical improvements.

We focused on short-term mortality without assessing long-term functional outcomes or quality of life. The institutional structure, featuring an intermediate care unit that diverts less severe cases, likely contributed to the higher observed mortality. The predominance of pneumonia (97.4%) limits the assessment of score performance across diverse infection sources. Among excluded patients, three with sepsis died within the first six hours of ICU admission. While a formal sensitivity analysis could not be conducted, this small number suggests that survivorship bias is unlikely to have materially affected the results, particularly as reliable calculation of APACHE II and SOFA scores within the first 24 h would have been unfeasible in this cohort. For patients discharged prior to day 28, vital status was confirmed at scheduled follow-up visits; deaths occurring outside the hospital before the follow-up appointment may not have been captured, although this is expected to represent a negligible proportion of the cohort given illness severity and the short follow-up interval. A detailed breakdown of exclusions by individual criterion was not prospectively recorded in the study database, which precludes the construction of a fully detailed patient flow diagram. Finally, a precise categorization of pneumonia by type (community-acquired vs. hospital-acquired) was not captured in the study database.

## Conclusion

APACHE II and SOFA demonstrated modest discriminative ability for 28-day mortality prediction in critically ill patients with sepsis, with comparable performance observed in the overall sepsis cohort (AUC 0.692 and 0.682, respectively; DeLong *P* ═ 0.608) and in septic shock (AUC 0.671 and 0.646, respectively; DeLong *P* ═ 0.828). The CCI exhibited acceptable discrimination in overall sepsis (AUC 0.667) but performed at chance level in septic shock (AUC 0.423, 95% CI 0.302–0.543, *P* ═ 0.195), likely reflecting the predominance of acute organ dysfunction over chronic comorbidity burden as the primary determinant of short-term mortality risk in this subgroup. The lower AUCs compared to larger heterogeneous populations indicate appropriate score behavior in a highly selected, severely ill cohort with high baseline mortality and near-universal mechanical ventilation, rather than inadequate performance. These findings support the continued clinical utility of conventional prognostic scores in high-acuity settings while emphasizing the critical importance of local validation and population-specific performance assessment prior to clinical implementation.

## Data Availability

Data supporting the findings of this study are available from the corresponding author upon reasonable request, subject to institutional ethics approval and data protection regulations.
